# The (mis)government in the COVID-19 pandemic and the psychosocial implications: discipline, subjection, and subjectivity

**DOI:** 10.1590/1980-220X-REEUSP-2021-0550

**Published:** 2022-03-14

**Authors:** Janaína Quinzen Willrich, Luciane Prado Kantorski, Ariane da Cruz Guedes, Carmen Terezinha Leal Argiles, Marta Solange Streicher Janelli da Silva, Dariane Lima Portela

**Affiliations:** 1 Universidade Federal de Pelotas, Faculdade de Enfermagem, Departamento de Enfermagem em Saúde Coletiva, Pelotas, Rio Grande do Sul, Brazil.; 2 Universidade Federal de Pelotas, Programa de Pós-Graduação em Enfermagem, Pelotas, Rio Grande do Sul, Brazil.; 3 Universidade Federal de Pelotas, Curso de Psicologia, Pelotas, Rio Grande do Sul, Brazil.; 4 Universidade Federal de Pelotas, Faculdade de Enfermagem, Programa de Pós-Graduação em Enfermagem, Pelotas, Rio Grande do Sul, Brazil.

**Keywords:** COVID-19, Remote Consultation, Mental Health, COVID-19, Consulta Remota, Salud Mental, COVID-19, Consulta Remota, Saúde Mental

## Abstract

**Objective:**

to analyze the psychosocial implications arising from the COVID-19 pandemic, reported in online service, from the perspective of Michel Foucault’s concepts of biopower, biopolitics and governmentality.

**Method:**

qualitative documental research, with analysis of medical records of users assisted in a therapeutic listening chat, between April and October 2020.

**Results:**

the data were organized into two themes: Governmentality in the COVID-19 pandemic and the production of psychosocial implications of anxiety and fear and Discipline and subjection in the COVID-19 pandemic: subjectivities marked by sadness and anguish. The first demonstrates that the “art of governing” in Brazil produced instabilities and uncertainties that influenced the production of fear of contamination/death/and non-access to treatment and anxiety. In the second theme, we can see how disciplinary control and biopolitical regulation are combined. In Brazil, an extremely unequal country, subjectivity and subjectivities marked by anguish, feelings of discouragement and sadness have been produced.

**Conclusion:**

the exclusionary processes were deepened in the pandemic, with the exercise of a biopolitics that makes life precarious and produces psychological distress.

## RESUMO

**Objetivo:**

analisar as implicações psicossociais decorrentes da pandemia da COVID-19, relatadas em atendimento online, pela ótica dos conceitos de biopoder, biopolítica e de governamentalidade de Michel Foucault.

**Método:**

pesquisa qualitativa do tipo documental, com a análise dos registros de prontuários de usuários atendidos em um chat de escuta terapêutica, entre abril e outubro de 2020.

**Resultados:**

os dados foram organizados em duas temáticas: Governamentalidade na pandemia de COVID-19 e a produção de implicações psicossociais de ansiedade e medo e Disciplinarizações e sujeições na pandemia de COVID-19: subjetividades marcadas pela tristeza e angústia. A primeira demonstra que a “arte de governar” no Brasil produziu instabilidades e incertezas que influenciaram na produção do medo da contaminação/morte/e não acesso ao tratamento e ansiedade. Na segunda temática, percebe-se como o controle disciplinar e a regulamentação biopolítica se combinam. No Brasil, um país extremamente desigual, tem-se produzido sujeição e subjetividades marcadas pela angústia, sentimentos de desânimo e tristeza.

**Conclusão:**

os processos excludentes foram aprofundados na pandemia, com exercício de uma biopolítica que precariza a vida e produz sofrimento psíquico.

## RESUMEN

**Objetivo:**

analizar las implicaciones psicosociales derivadas de la pandemia de la COVID-19, relatadas en el servicio online, desde la perspectiva de los conceptos de biopoder, biopolítica y gubernamentalidad de Michel Foucault.

**Método:**

investigación documental cualitativa, con análisis de prontuarios de usuarios atendidos en chat de escucha terapéutica, entre abril y octubre de 2020.

**Resultados:**

los datos fueron organizados en dos temas: Gubernamentalidad en la pandemia de la COVID-19 y la producción de implicaciones psicosociales de la ansiedad y el miedo y Disciplinas y sujeciones en la pandemia de la COVID-19: subjetividades marcadas por la tristeza y la angustia. El primero demuestra que el “arte de gobernar” en Brasil produjo inestabilidades e incertidumbres que influyeron en la producción de miedo a la contaminación/muerte/y no acceso al tratamiento y ansiedad. En el segundo tema, podemos ver cómo se combinan el control disciplinario y la regulación biopolítica. En Brasil, país sumamente desigual, se han producido subjetividades y subjetividades marcadas por la angustia, sentimientos de desánimo y tristeza.

**Conclusión:**

los procesos de exclusión se profundizaron en la pandemia, con el ejercicio de una biopolítica que precariza la vida y produce sufrimiento psíquico.

## INTRODUCTION

Throughout history, different epidemics and pandemics have had a devastating effect on people’s physical and mental health. The challenges imposed by the current reality of the COVID-19 pandemic are complex, pointing to the effects of several orders.

Over the years, humanity has faced diseases such as AIDS, Ebola, Severe Acute Respiratory Syndrome (SARS) and now COVID-19. Contagious diseases continue to threaten human populations and historians continue to look to the past for insights to understand the present^(1)^.

In *La Peste*, the author sought to organize the archetypal structure of an outbreak, describing the social drama triggered by an epidemic and explaining the latent social structures, being conducive to the analysis of the social context^(2)^. The fear of contracting the disease and of infecting, and emotional responses to the pandemic, such as inappropriate behaviors, emotional distress, defensive manifestations, anxiety, frustration, loneliness, anger, boredom, depression, stress and avoidance behaviors, are found in several studies^(3,4,5,6)^.

Social isolation, recommended by the World Health Organization (WHO) as a health measure to reduce contagion, and the circuit of fear that is established allow us to resume the faces of Foucault’s governmentality, which aims to control and manage human life in its multiplicity (population), intervening on the possibilities of individuals to act in order to conduct their conduct^(7)^. However, we experience in Brazil contradictory discourses in relation to isolation, being proposed and implemented by the states, but constantly attacked by the central government, which defended economic interests to the detriment of the defense of life.

Throughout the COVID-19 pandemic, the mobilization of strategies for promotion, prevention and care in mental health and psychosocial support has become one of the priorities described by the WHO. Faced with isolation and the need to minimize one-to-one interaction, online mental health services present themselves as an important resource, in the sense of facing the sense of uncertainty and fear^(6)^, as well as an ally in the regular assessment of symptoms of depression, anxiety, stress and suicide risk^(8,9,10)^.

In this modality of digital mental health care, already used before the COVID-19 pandemic in countries in the Middle East and North Africa^(10)^, and developed in Brazil by the Federal Nursing Council (COFEN – *Conselho Federal de Enfermagem*)^(11)^, therapeutic communication has been confirmed as a powerful tool for the reception of challenging experiences, due to its ability to produce reflections that allow the organization of individual and collective reality^(12)^.

In this way, this article aims to analyze the psychosocial implications resulting from the COVID-19 pandemic, reported in online service, from the perspective of Michel Foucault’s concepts of biopower, biopolitics and governmentality.

## METHOD

### Design of Study

This is a qualitative, descriptive, document analysis type research.

### Population

A total of 81 people were assisted in a therapeutic listening chat from April to October 2020.

### Local

Therapeutic listening channel, available on the website of the Research Group in Nursing, Mental Health and Collective Health, linked to the Brazilian National Council for Scientific and Technological Development (CNPq – *Conselho Nacional de Desenvolvimento Científico e Tecnológico*), available at www.gruposaudemental.com/chat.

### Selection Criteria

Agree to participate in the study, by signing the Informed Consent Form, and be 18 years of age or older.

### Data Collection

Data collection was carried out in the electronic medical records of users served in the chat, between April and October 2020. The records included sociodemographic data, history, reported complaints, evolution of care, intervention and planning in teleservices. The methodological procedures followed the COREQ (COnsolidated criteria for REporting Qualitative research) guidelines. The checklist covered everything from the manuscript design phase to the presentation of results.

Twenty-six professionals participated in the therapeutic listening action, being 23 operators in direct service to chat users, two in the technical team and three supervisors, two of which also acted as operators. The team was made up of nurses, psychologists and occupational therapists, 24 women and two men, who received training prior to the start of care. During the activities, there was weekly supervision in small groups and thematic workshops to update the team. For the appointments, a weekly schedule was planned, in which professionals voluntarily assisted on shifts.

### Data Analysis and Treatment

Qualitative data from electronic medical records were analyzed, based on Michel Foucault’s concepts of biopower, biopolitics and governmentality.

### Ethical Aspects

The research was approved by the Institutional Review Board of the Faculty of Medicine at the *Universidade Federal de Pelotas*, under Opinion 4,003,186 of 2020. All participants agreed to participate through signing the Informed Consent Form. Ethical aspects were protected in accordance with Resolution 466 of 2012 of the Brazilian National Health Council (*Conselho Nacional de Saúde*)^(13)^. Therapeutic listening via chat complied with the guidelines of COFEN Resolution 634 of 2020, which provides for the authorization of nursing teleconsultation as a way of combating the COVID-19 pandemic^(14)^. The records are analyzed in this article and identified by the letter C for “care” and a coding number (C1, C2…).

## RESULTS

The records of care performed between April and October 2020 were analyzed in the electronic medical records. There were 81 people assisted, and the number of care per person ranged from 1 to 52. However, there was a predominance of care for 56 people, configuring a punctual listening; 22 people were seen between 2 and 8 times; 3 people demanded more continuous care, between 22 and 52 times. Most care (48) lasted between 31 and 60 minutes.

### Governmentality in the COVID-19 Pandemic and the Production of Psychosocial Implications of Anxiety and Fear

This theme brings reports that point out how the “art of governing” in Brazil, producing instabilities and uncertainties that influenced the production of anxiety, fear of contamination/death/and lack of access to treatment, as shown in the records that follow.

*She relates anxiety attacks to the current pandemic scenario, says that she is “haunted by the virus” and that any symptom, regardless of being a flu symptom, makes her suspect that it may be the manifestation of COVID-19. In addition, the uncertainty about her work situation due to the pandemic bothers her. (C55)*

*She tries to control herself as much as possible, but the news that there are cases of COVID-19 in her condo has destabilized her, she associates this with other things that are happening in her life. She points out that we are going through a difficult time, but when she gets “closer to us” it seems that it gets more complicated! (C59)*

*She reports concerns related to the pandemic, complies with all protocols, however she is anxious when her father who works returns to the house where they live, has not seen her boyfriend for more than a month because he also works, says she is very afraid of losing someone she loves. (C3)*

*She is worried that the city has passed to a red flag, she is afraid of getting sick and not having a bed, of contaminating her family members. She presents symptoms of anxiety and fear, recognizes symptoms and sought help in chat. (C9)*

*She says that she sometimes sleeps little, that she is anxious, controlling and is afraid that something might happen to the people she loves. (C33)*

*She’s very anxious. She is afraid of losing her job. She is very mobilized with the situation of the pandemic, afraid to go out on the street, go to the lottery, the market and need to go out daily to work. (…) she reports having (1 to 2 times a week) shortness of breath, light sweating, sometimes dizziness, hyperventilation at night. (C44)*

### Discipline and Subjections in the COVID-19 Pandemic: Subjectivities Marked by Sadness and Anguish

In this theme, the data demonstrate how disciplinary control and biopolitical regulation combine, and in Brazil, an extremely unequal country, subjectivity and subjectivities marked by anguish, feelings of discouragement and sadness have been produced.

*She says that in the last 3-4 months she has been finding it constant to be difficult to carry out her activities and get out of bed, which later generates guilt. She can identify that the context of the pandemic significantly influenced the issue of distancing people. (C8)*

*She reported feeling sad on the day, but without associating it with any triggering event. She says she doesn’t usually feel that way. (…) in the current context of intense coexistence, she has tried to get away to avoid conflicts, a fact that she believes leaves her father hurt. (C23)*

*She mentioned feeling affected by the news she follows daily about the country and that this directly influences her mood and her desire to isolate herself. (…) she was having a lot of difficulty talking during the entire service, crying a lot due to the impossibility of visiting her parents in another city. (C57)*

*Now, with social isolation, the symptoms are more intense, she has fear, shortness of breath, thoughts that something bad can happen and insomnia, she also says that she respects the distance, but that seeing other people disrespecting her brings a bad feeling. (C58)*

## DISCUSSION

Since its emergence in late 2019 in Wuhan, China, the new coronavirus has left a trail of deaths and infections, eventually producing significant changes in ways of living, as more than half of the world’s population has been oriented towards social distancing as well as changing everyday habits such as hand hygiene and respiratory hygiene in order to slow the spread of the SARS-CoV-2 virus.

However, humanity has already faced other pandemics, such as the bubonic plague and the Spanish flu, as described in *O Nascimento da Clínica*, in which, due to similar characteristics to the current COVID-19 pandemic, measures similar to the current ones have been taken, such as social distancing, quarantine or lockdown. These measures demonstrate the articulation between medical knowledge and public health policies, which created a biopolitical strategy for managing the lives of individuals who make up a population, which is in the order of medical, organizational and political knowledge of a State.

In the current pandemic context, a biopolitical strategy of epidemic medicine stands out, defined by a political statute of medicine and a medical conscience at the State level, which is responsible for the constant task of informing, controlling and coercing, comprising objects related to the police, and medical competence^(15)^. Living in this context of psychosocial risk, in which one lives with the fear of contamination and death and subjection to the current biopolitical strategy that altered social relations, has produced implications.

Here, we highlight the understanding of psychosocial implications as the phenomenon related to the way in which individuals experience these social and collective conditions of psychosocial risk resulting from the COVID-19 pandemic, from negative effects at a psychological, physical and social level, expressing in the affective and behavioral dimensions in the form of stress, exhaustion or depression.

Two questions are a starting point to analyze the records of online care records carried out during the COVID-19 pandemic period. In the first, the psychosocial implications are not considered as diseases, but as expected effects in a situation of psychosocial risk. In the second, the analysis made here based on the concepts of power analytics in Foucault, proposing to contextualize the psychosocial implications of the COVID-19 pandemic in the current historical scenario, not to criticize the current functioning of disciplinary control and biopolitics as technologies of power. It is understood, as Foucault, power as positive, because, when it is imposed, it is also constructed by individuals, cellularly and collectively, transforming itself in the course of historical events from factors that combine unpredictable external and internal movements that subject, but also subjectivize as it is assumed by those involved.

### Governmentality in the COVID-19 Pandemic and the Production of Psychosocial Implications of Anxiety and Fear

The investigation of the subject not only subjected to practices of coercion, but conducting itself in practices of freedom, became the focus of Foucault’s analysis in the field of power relations, from 1978 onwards, when the notion of governmentality, or government, came to have a central place in Foucault’s research, leading to a certain turn in his theoretical and political positioning, which placed the subject, the truth and the constitution of the experiences of themselves and others^(16)^.

It is in this conceptual perspective of government, in a broader sense, that both the State’s political and management structures and the way of managing individuals’ and groups’ conduct are designated. Foucault mentions subjectivity not only as submission, but as a mode of counter-conduct to power, whose action enables resistance or a search for “how to become a subject without being subjected”^(17)^.

In 1981, *Subjetividade e Verdade* points to a new sense of relationship with us beyond our own individuality, since we are constituted by the relationships we have with others, because the self practices are social practices and in the relations of power there remains the joint coexistence, from antiquity to modernity, subjection practices and self practices^(16)^. From this new meaning, the relevance of analyzing the psychosocial implications of the COVID-19 pandemic stands out, which are experienced subjectively, but inserted in collective and social events. The first issue identified in the electronic medical records showed that the subjects are experiencing anxiety resulting from the fear of contamination by the coronavirus, fear related to the difficulty of accessing health services and fear regarding the work and income situation.

These aspects, pointed out by the subjects assisted in the chat, are closely influenced by the management of the pandemic in Brazil. To analyze this management, from the perspective of Foucault’s governmentality, points to a (mis)government, i.e., the absence of actions based on principles, tactics, calculations and specific knowledge that would enable the Brazilian State to govern the COVID-19 pandemic in a rational and thoughtful manner.

The SARS-CoV-2 virus brought the threat of death, making the pandemic a condition of possibility for the adoption of measures to preserve the population’s lives worldwide. Unlike the exercise of sovereign power, which had the right of life and death over its subjects and operated by causing to die and letting live, biopower functioning is a way of governing life, converting the power of death into a power that is exercised positively over life, interfering in its management, in its increase and in its multiplication based on precise controls and overall regulations^(18)^.

This movement in favor of life led political power to take on the task of managing people’s lives through discipline and biopolitics, i.e., biopower is constituted from two poles, which are the body discipline (disciplinary power) and the population regulations (biopolitics). It is a “two-sided technology – anatomical and biological –, individually and specifically, focused on the body performance and facing the processes of life, it characterizes a power whose highest function is no longer to kill, but to invest in life, in a top to bottom”^(18)^.

Biopower, in its disciplinary and biopolitical technologies, operating in the COVID-19 pandemic world, is exercised in a distinct and unprecedented context, since this pandemic differs from the others faced by humanity in at least two issues. First, in relation to death as a private and individual ritual, before the pandemic, it becomes collective from successive funeral rituals of global scope without, however, the physical presence of family members, imposing the collective meaning of death on the particular mourning. With the daily count of the dead, the pandemic has made death entirely dominate life^(19)^. The second issue that crosses the pandemic context and makes it unique is network connectivity. In other words, information and communication technology connects individuals and puts them in front of events in real time, such as the lack of hospital beds around the world, in addition to the hundreds of daily deaths being collectively experienced, changing the way of dealing with losses and grief itself^(20)^. It is noticed that the people assisted in the chat, when experiencing this context, which connects individual and collective tragedies, have presented anxiety due to fear, contamination/death, difficulty in accessing health services, loss of family members and loss of income.

In Brazil, in addition to the issues of changes in relation to death/bereavement and network connectivity, which makes difficulties experienced in real time by the entire world population, the emergency period has been marked by the difficulty of articulation between different social actors and competing competence among the federated entities in the matter of collective health. The “art of governing”, marked by the absence of a directive from the central government and by the alternation in the main management positions in the Ministry of Health, implied a “low performativity of governance and in the inexpressive capacity of articulation between the other spheres”^(21)^.

This disarticulation of the Ministry of Health culminated in the “difficulty of producing reliable health guidelines, the lack of resources and the certain delay in disseminating information in the national territory”, in addition to the operation under the aegis of a vertical deregulation with manifestations “in disagreement with the protocols established by the World Health Organization” and the strategy of the low number of tests, which made it difficult and difficult to estimate the real pandemic^(21)^.

This context of instability and uncertainty, in the midst of a pandemic of catastrophic dimensions, which produced the collapse of the hospital system around the world due to the high transmissibility of the disease and the need for hospitalization and Intensive Care Unit (ICU) support for part of those infected^(22)^, directly influences the production of fear of contamination/death/and non-access to treatment, reported by people who were assisted in the chat. Tension affects a large part of the population, whether infected or not, and fear intensifies stress and anxiety levels in healthy people^(23)^. However, it is known that people react to adversity in unique ways, and the reactions are influenced by factors such as the nature and severity of the event to which they were exposed, and how vulnerable the person is at the moment. It is important to note that this reaction will not necessarily be defined as a disease, as most will be a normal reaction to an atypical situation^(24)^.

The pandemic consequences go far beyond mortality or possible after-effects of the disease and implications for the health system as a whole, as they also affect the economy, social relationships and the environment. Economic impacts are strictly related to the fear of job and income loss, reported in the excerpts presented, resulting from the social distancing, quarantine and lockdown measures associated with the pandemic.

Isolation and quarantine are two terms often used with the same meaning, but they have applicability in different situations. Isolation is the separation of contaminated people from uncontaminated people. Quarantine is the restriction of activities or the separation of people who do not have the disease, but may have been exposed to the infectious agent, in order to monitor and detect the disease early. The effectiveness of isolation decreases due to transmission being possible before the symptomatic period and the difficulty in isolating and tracing cases and contacts. Quarantine, which also depends on case detection and contact tracing, has good effectiveness when carried out in a short term and quickly^(22)^.

In a scenario in which it is no longer possible to identify the infected and their contacts, community containment measures are necessary to slow the spread of the disease. This is a type of intervention intended to reduce interactions and movements between people, except for minimal interaction, in order to guarantee basic supplies. It ranges from social distancing measures (closing schools and canceling public events) to completely blocking activities in a city (lockdown). Community control can occur from the suppression strategy (horizontal social isolation) or mitigation strategy (vertical social isolation)^(22)^.

Regarding isolation, in Brazil there was a mismatch of information between the Ministry of Health and the Presidency of the Republic, producing a discursive daily life of tension. The Ministry, following WHO recommendations, recommended the widespread social isolation of all Brazilians so that transmission could be distributed over a longer period of time and the hospital back-up was guaranteed for everyone who needed it. The Presidency of the Republic, on the other hand, stated that isolation should be carried out only by infected people and those who are in risk groups, with the argument that horizontal isolation would produce an economic crisis, with an increase in unemployment and misery. However, it is known that vertical isolation is not effective in protecting people at risk of developing serious illness and death, since, even with the restriction of circulation of these risk groups, they may still have contact in the home environment with individuals who leave the house and, therefore, are more exposed to the virus^(22)^.

Horizontal isolation measures seem to be something out of time, given the scientific advance, the progress of information and communication technologies. However, the COVID-19 pandemic reveals that these measures, used in past pandemics, are the only effective ones for their control. In this way, it is possible to establish a relationship between COVID-19 and the plague declared at the end of the 17^th^ century, and which was described in *Vigiar e Punir* ^(7)^. In the book, disciplinary schemes raised by the plague are described, which consisted of strict spatial policing (suspension of circulation (which will only occur when strictly necessary and in shifts), multiple separations and individualizing distributions), constant inspection and permanent surveillance, determining an intensification and ramification of a power that multiplies, articulates and is subdivided, crossing the city through hierarchy, surveillance, through the look and through documentation.

The biopolitics exercised in the Brazilian scenario, marked by the lack of concrete policies that guarantee horizontal isolation measures, produced the precariousness of lives and made Brazilians live the dilemma of fear of exposure to the virus and the need to work, a fact reported by people answered in the chat. The production of this “economic man” in contemporary times has potentiated “the governmentality of lives under the aegis of a state that is absent and incapable of distributing wealth and minimizing inequalities”^(21)^.

### Discipline and Subjections in the COVID-19 Pandemic: Subjectivities Marked by Sadness and Anguish

It is observed that the COVID-19 pandemic is placed as a beam in which the mechanisms of power technology are present, to the extent that disciplinary control and biopolitical regulation combine. In Brazil, an extremely unequal country, this combination has produced subjection and subjectivities marked by real effects that place on the population the responsibility to go through this period of sanitary emergency. This has produced suffering and anguish, since the subjects find themselves alone facing all the crises arising from the pandemic, such as health crisis (increase in the number of deaths and collapse of the health system), economic crisis (unemployment, increased cost of living) and social crisis (affected social relations).

In coping with these crises individually, especially social crisis, in which social relations were affected by the pandemic and social distancing measures, work relationships were modified, friendship relationships were broken or had decreased interactions and family interactions were intensified (between residents of the same house) or impaired (in case of family members in the risk group). Moreover, the relationship with themselves was modified, since the subjects are placed in front of themselves, with greater space for reflection in everyday life, and greater encounter with their fears and weaknesses, with the prospect of death and illness being prominently present. These changes in the way they relate to themselves and others, as reported by the subjects assisted in the chat, produced feelings of discouragement, sadness and decreased energy.

The pandemic caused by the SARS-CoV-2 virus points to a certain indistinction in the functioning of disciplinary power and biopower, since the protection of individual and collective life merges simultaneously, blurring the dichotomy that prevails in the West between individual and collective interests. Thus, an individual attitude affects the other and the collective, justifying the government’s individual actions based on disciplinary power, as they have relevance in the biopolitical regulation of populations at the global level^(20)^.

However, in the Brazilian State’s governmentality, statistical science, the basic tool for understanding biopower regulation strategies, points out, from epidemiological data, the impacting coronavirus victim numbers in Brazil and reveal the ineffectiveness of health management by the central government^(20)^. Therefore, we experience a discursive daily life marked by tension between public statements of rulers and scientific studies. At all times, there is a dispute between discourses, in favor of social isolation, in defense of the Unified Health System (SUS – *Sistema Único de Saúde*) and science. Those who report false data advocate the dismantling of public policies, “discredit in science and a nod to prophetic hysteria”^(21)^.

The discourse produces “truths”, and as it is the object of appropriation and manipulation, ends up assuming large dimensions in times of social networks, because it begins to determine and articulate the production of new discourses, new truths that reach the subjects and produce effects that affect their subjectivity. The important thing in this matter of truth is that certain things actually seem to be true and that the subjects must either produce them personally, accept them or submit to them. Therefore, what has been and will be in question is truth as a bond, as an obligation, as a policy, and not truth as the content or formal structure of knowledge^(15)^.

The way in which subjectivities and self-experiences are constituted from the obligations of truth demonstrates how the government operates on subjectivity, making it calculable, thus enabling a preponderantly psychological self-regulatory technique capable of mapping all our “private selves”^(21)^.

It is customary to think of power as something outside the subjects, which pressures them and submits them to an order. However, from Foucault, power began to be understood as something positive that produces subjects with their desires and in their conditions of existence, i.e., power is not only what we must resist or owe, but also what we shelter and depend on to exist^(25)^.

We experience palliative society, which coincides with the society of neoliberal performance, in which negativities such as prohibitions and punishments (typical of disciplinary societies) are replaced by positivity such as motivation, self-optimization or self-realization. “Be happy is the new formula of domination”, which is exercised without any effort, because the subjects are assumed to be free and not aware of their submission. “Freedom is not repressed, but exploited”^(19)^.

In the context of a pandemic, the subjects are called to exercise the “freedom of choice”, such as not isolating themselves, not being vaccinated, not wearing masks, in short, discourses that work to subjectively affect social subjects. Under this logic, the message expressed by the Brazilian government management ceases to place itself as a state of protection to act as a predatory state, i.e., it plays with the prospect of possible death of part of the population. In the pronouncement of the phrase “Some will die”, uttered by the Brazilian head of state, the State’s necropolitical management^(26)^ during the COVID-19 pandemic, which imposes different actions in relation to certain groups, such as forced exposure of the most vulnerable, dictating the differential distribution of the right to life, marked, among other aspects, by the interest of capital.

Thus, anchored in Foucault’s theories, it is recognized that there is a fabrication of life, a manufacture of subjects “capable of bearing the burden sums of freedom in contemporary Western life forms”^(21)^. “Psy” subjects were manufactured by a positive psychology that deals with happiness, well-being and optimism, which undergo the logic of neoliberal performance. The device of happiness individualizes the human being, depressing them, since they put suffering, for which society would be responsible, as something privatized, psychologized and resulting from individual failure, in addition to producing the depoliticization and desolidarization of society^(27)^.

The disciplinary society is the order of negativity, which produces madmen and delinquents, different from the performance society, which “produces depressives and losers”. Depression is the disease of a society that suffers excess of positivity, work and performance that culminates in self-exploitation^(27)^. This feeling of failure, feeling of not taking care of tasks or not achieving a good performance in performing daily activities generates guilt and produces suffering, as can be seen in the reports of people assisted in the chat.

### Study Limitations and Advances in Nursing/Health

As a study limitation, it is emphasized that, as it is a very current and still ongoing theme, there are no robust studies that allow comparations and counterpoints. As advances for health and nursing, the main contribution is the possibility of analyzing, based on Foucault’s concepts, suffering and psychosocial implications in a social, collective and political perspective. The creation of a therapeutic listening chat linked to the Faculty of Nursing guaranteed social commitment at the time of a pandemic.

## CONCLUSION

The analysis of electronic medical records of online care, based on Foucault’s concepts, allowed a reading of the pandemic context and how it has affected the subjects to go through daily life, in which family, social and work experiences were unfolded in this period of pandemic and social distancing. Psychosocial implications, such as fear of coronavirus contamination and loss of work and income, anxiety, sadness and anguish in relation to future expectations are closely marked by the exercise of governmentality and a biopolitics that demonstrate a total devaluation of life by the Brazilian State. In addition to the denial of the severity and lethality of the virus, the absence of concrete measures to guarantee isolation and the necessary income for a dignified life produced the precariousness of lives. The exclusionary processes in the country, which is extremely unequal, have been deepened with the pandemic. Instead of prioritizing the collective over individualities, once the health of one subject interferes with the health of the other and the community, Brazilians were left to fend for themselves in a smokescreen that puts all disrespect for life and political incompetence as respect for individual freedoms.

## ASSOCIATE EDITOR

Divane de Vargas
